# Comparison of Models for IP_3_ Receptor Kinetics Using Stochastic Simulations

**DOI:** 10.1371/journal.pone.0059618

**Published:** 2013-04-10

**Authors:** Katri Hituri, Marja-Leena Linne

**Affiliations:** Computational Neuroscience Laboratory, Department of Signal Processing, Tampere University of Technology, Tampere, Finland; SUNY Downstate MC, United States of America

## Abstract

Inositol 1,4,5-trisphosphate receptor (IP_3_R) is a ubiquitous intracellular calcium (Ca^2+^) channel which has a major role in controlling Ca^2+^ levels in neurons. A variety of computational models have been developed to describe the kinetic function of IP_3_R under different conditions. In the field of computational neuroscience, it is of great interest to apply the existing models of IP_3_R when modeling local Ca^2+^ transients in dendrites or overall Ca^2+^ dynamics in large neuronal models. The goal of this study was to evaluate existing IP_3_R models, based on electrophysiological data. This was done in order to be able to suggest suitable models for neuronal modeling. Altogether four models (Othmer and Tang, 1993; Dawson *et* *al.*, 2003; Fraiman and Dawson, 2004; Doi *et* *al.*, 2005) were selected for a more detailed comparison. The selection was based on the computational efficiency of the models and the type of experimental data that was used in developing the model. The kinetics of all four models were simulated by stochastic means, using the simulation software STEPS, which implements the Gillespie stochastic simulation algorithm. The results show major differences in the statistical properties of model functionality. Of the four compared models, the one by Fraiman and Dawson (2004) proved most satisfactory in producing the specific features of experimental findings reported in literature. To our knowledge, the present study is the first detailed evaluation of IP_3_R models using stochastic simulation methods, thus providing an important setting for constructing a new, realistic model of IP_3_R channel kinetics for compartmental modeling of neuronal functions. We conclude that the kinetics of IP_3_R with different concentrations of Ca^2+^ and IP_3_ should be more carefully addressed when new models for IP_3_R are developed.

## Introduction

Inositol 1,4,5-trisphosphate receptor (IP_3_R) is a ligand-gated calcium (Ca^2+^) release channel typically expressed on the endoplasmic reticulum (ER) in neurons and many other cell types. It has a major role in intracellular Ca^2+^ dynamics which, in turn, is involved in many cellular processes such as muscle contraction, neurotransmitter release, vesicle secretion, fertilization, gene transcription, immunity, and apoptosis. In neurons, dynamical changes in Ca^2+^ concentration ([Ca^2+^]) are involved, among others, in neuroplasticity and development (see recent reviews [1], [2]), and in neurodegeneration (see [3], [4]). Transient, repetitive changes in cytosolic Ca^2+^ concentration are crucial for synapse modification and plasticity, including long-term potentiation (LTP) and long-term depression (LTD) [5]–[8]. These phenomena constitute the biological basis for learning and memory formation in the brain [8], [9]. Particularly in the cerebellum, IP_3_Rs are relatively highly expressed in Purkinje cells [10]. Ca^2+^ release from ER has been shown to be a key mediator of cerebellar LTD [11].

The inositol 1,4,5-trisphosphate receptor is a tetrameric receptor-channel, consisting of four sub-units. In total, three different genes (ITPR1, ITPR2, and ITPR3) encode three different types (1, 2, and 3) of IP_3_R and their splice variants from which homo- or heterotetramers can form [12]. IP_3_R is activated and opened by both IP_3_ and Ca^2+^. Ca^2+^ can also act as the inhibitor of IP_3_R in higher concentrations. IP_3_ is produced from phosphatidylinositol 4,5-bisphosphate (PIP) by phospholipase C (PLC). After a cell is stimulated (for example by glutamate in neurons) certain G protein- or tyrosine kinase-linked receptors are activated. These, in turn, can activate PLC. ER acts as a Ca^2+^ store, and while open, IP_3_R can release Ca^2+^ from ER lumen to the cytosol. Transient rises or oscillations in Ca^2+^ concentration can then activate various enzymes and even induce changes in the transcriptional level. IP_3_Rs are known to be responsible for the phenomenon called Ca^2+^-induced Ca^2+^ release (CICR), in addition to ryanodine receptors (RyRs) [13], [14].

In order to develop models for ion channels and receptors detailed data on the structure and function of the modeled entity is required. The function of IP_3_R has been studied with electrophysiological techniques. However, since IP_3_Rs are prevalently located on the endoplasmic reticulum of a cell, performing the recordings is not straightforward. The first recordings performed on IP_3_Rs involved isolated microsomes from smooth muscle cells incorporated into artificial lipid bilayer [15]. Later, the same technique has been used, for example, for IP_3_R in canine cerebellum [16]–[20], in mouse cerebellum [21], and in HEK cells [22] (IP_3_R recombinantly expressed). IP_3_Rs have also been recorded from the plasma membranes of DT40 cells [23] (IP_3_R endogenously expressed (native)) and DT40-3KO cells [24], [25] (stably expressed IP_3_R construct, native IP_3_R ablated). Since the nuclear membrane is a continuation of the ER, IP_3_Rs have also been recorded from isolated nuclei of *Xenopus* oocytes (for example [26] (recombinantly expressed and native IP_3_Rs), Purkinje neurons and granule cells [27], [28] (IP_3_R endogenously expressed), and DT40 cells [23], [29]. These kind of data are of great value when developing a model for ion channel kinetics. However, the electrophysiological raw data on IP_3_R is not available in any of the publicly available databases, but its statistics is described in publications. For example, the dependence of open probability on cytosolic Ca^2+^ or IP_3_ concentrations is given ([16], [19], [20], [29], [30]). In some cases, the open and closed time distributions [18], [20], [22] or mean open time [17], [18], [22], [29] are also reported. In an ideal case, the raw data would be publicly available in a database and a modeler could extract all needed statistical measures out of the data or use the raw data for automated estimation of model parameter values.

In addition to electrophysiological measurements, Ca^2+^ imaging and radioactive assays have also been used to study the behavior of IP_3_R *in vitro*. For example, Fujiwara *et al.* [31] analyzed the kinetics of Ca^2+^ release via IP_3_R in controlled cytoplasmic environment in permeabilized cerebellar Purkinje cells. In addition, superfusion and ^45^Ca^2+^ release assay (radioactive assay) have been used for studying the Ca^2+^ release and inhibition of IP_3_R by Ca^2+^ in hepatic microsomes [32]–[34]. These kind of studies give more detailed information on the IP_3_R regulation by IP_3_and Ca^2+^ and their affinities than electrophysiological studies. In some cases, the data obtained from Ca^2+^ imaging studies or from radioactive assays has been used in modeling studies, for example Fujiwara *et al.* [31] by Doi *et al.* [32] and Dufour *et al.* [35] by Sneyd *et al.* [36].

In order to reach a better understanding of the dynamical behavior of IP_3_R, as well as its involvement in various cellular processes, it is of interest to build models of IP_3_R. Computational models are important for understanding the time evolution, dynamics, and regulation of ion channels and intracellular proteins and enzymes [37], [38]. Several models have previously been proposed to describe the behavior of IP_3_R (for a comprehensive review, see, for example [39]). There are models presented for different types of IP_3_R (type 1, 2, and 3) [12] in different animals, tissues and cells (for example *Xenopus* oocyte [40], cerebellar cells [41], pancreatic acinar cells [42], and hepatic cells [32]). The first and most well-known model is the one by De Young and Keizer [41]. Some models for IP_3_R have been compared either analytically or by means of simulation [36], [43]–[45], and later reviewed [39], [46].

The majority of the existing models is deterministic. Deterministic approaches, however, do not give biologically valid results and are not always capable of modeling the random behavior observed with small numbers of molecules [47]–[50]. Stochastic modeling is therefore more and more used for describing the dynamics of a biochemical system. The stochastic approach is always valid whenever the deterministic approach is valid, but when the deterministic is not, the stochastic might sometimes be valid [51]. Most commonly, deterministic methods and, in some cases, analytical methods are used to investigate the properties of IP_3_R models (see, for example [43] or [52]). More rarely, stochastic methods are applied [53], [54], even though it is known that the behavior of ion channels is stochastic.

Despite the wealth of IP_3_R models the selection of a specific model for describing IP_3_R related calcium dynamics or signaling is not straightforward. The models are seldom generic in nature and capable of describing all possible data obtained for a specific IP_3_R or cell type. The reason for this is that the models are developed for some specific purpose, describe the behavior only in certain experimental conditions, or the dynamics are not fully analyzed to validate the model. This can be due to the limited access to experimental data. We therefore wanted to study the dynamics of existing models in detail and to specifically address their suitability in the context of complex neuronal models. In this work, the interest is set on the type 1 IP_3_R because it is most commonly expressed in neurons [10]. After a preliminary study, we chose four models [35], [55]–[57] for a more detailed analysis and comparison. Other models did not meet our criteria. The chosen models were originally developed by using data either from IP_3_R in canine cerebellum or type 1 IP_3_R. As the selected models are biophysically realistic and based on the law of mass action, they can be implemented to the stochastic simulation tool STEPS [58], [59] used in this study. Additionally, we decided to concentrate on computationally inexpensive IP_3_R models so that it would be possible to integrate them as part of larger model for calcium dynamics or synaptic plasticity. We validated the functionality of the models by comparing the statistical behavior of IP_3_R channel kinetics (open probability curves, mean open times, and open and closed time distributions) to the equivalent obtained by electrophysiological recordings from IP_3_Rs expressed in neurons.

Our results show firstly, that the behavior of the studied models varies in similar simulation conditions and, secondly, some models show quite unrealistic kinetic behavior. We therefore conclude that the kinetics of IP_3_R (open and closed times and the open probability) with different concentrations of both Ca^2+^ and IP_3_ should be more carefully addressed when new models for IP_3_R are developed.

## Materials and Methods

In our present work, after a preliminary review on existing IP_3_R models, we selected four models [35], [55]–[57] for comparison. The selection was based on the following criteria: (1) relative simpicity (i.e. the model should have less than 20 states), (2) development based on data obtained from neuronal or type 1 IP_3_R, and (3) basis in the law of mass action (the reactions include binding and unbinding reaction and state transitions). As our ultimate goal is to find a model that can be an integral part of a larger model for Ca^2+^ dynamics or synaptic plasticity in neurons, it is an advantage to have a structurally simple model. The selected models are based on the law of mass action and can thus be implemented into the stochastic simulators such as STEPS [58].

### Models

#### The model of Othmer and Tang

**Figure 1:**
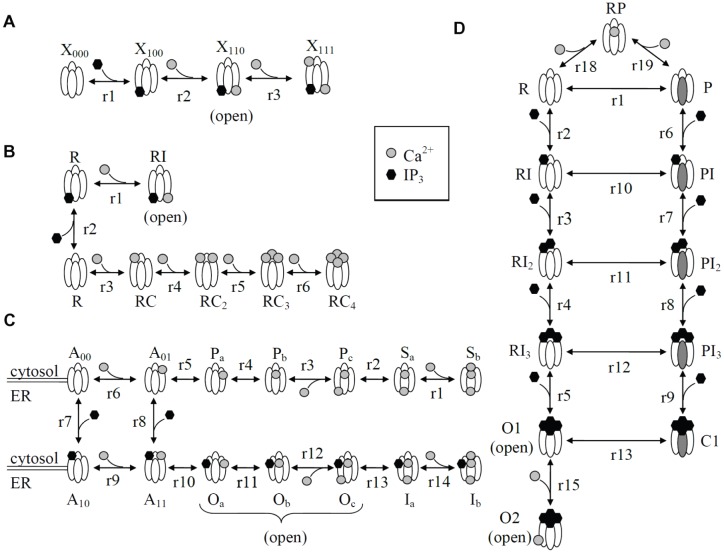
Schematic representation of the states and transitions of the IP_3_R models. (A) Othmer and Tang [55] (forward direction of a reaction is to the right) (B) Doi *et al.* [35] (forward direction of a reaction is to the right or up), (C) Fraiman and Dawson [57] (forward direction of a reaction is to the right or down) (D) Dawson *et al.* [56] (forward direction of a reaction is the to the direction of binding a ligand or in the plain state transitions from left to the right).

The model of Othmer and Tang [55] is one of the earliest and small- scaled models regarding the number of states. There is only four states, since the binding order of Ca^2+^ or IP_3_ is not free, but sequential, opposite to the models of De Young and Keizer [41] or Bezprozvanny and Ehrlich [18]. Othmer and Tang [55] assume that IP_3_ has to bind to its binding site before Ca^2+^ can bind and the channel can open, as well as the activating Ca^2+^ has to bind to its site before the inhibition by Ca^2+^ can occur. The schematic representation of the model of Othmer and Tang [55] in Figure 1A and the parameter values in Table 1 were used in this study. The model of Othmer and Tang [55] has been used before as a part of a larger model for calcium dynamics, for example, by Mishra and Bhalla [60].

**Table 1:** Rate constants for IP_3_R model of Othmer and Tang [55].

Reaction	*k_f_*	*k_b_*
r1	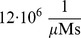	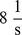
r2	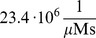	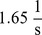
r3	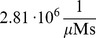	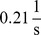

r1 to r3 refer to reactions represented in Figure 1A.

#### The model of Dawson *et al*

Dawson *et al.* [56] built a model for IP_3_R, using a RyR model by Sachs *et al.* [61] as their starting point, to understand the adaptive and incremental behavior of IP_3_R. The model of Dawson *et al.* [56] is applicable to type 1 and 2 IP_3_Rs and with some modification to type 3. Dawson *et al.* [56] assume that IP_3_R has two conformations, R and P. The conformation R can bind four IP_3_molecules rapidly, but with low affinity, to reach an open state. The conformation P, on the other hand, slowly binds four IP_3_ molecules, but with high affinity, to reach a closed state where it is thereafter possible to reach the open state. In this work, Scheme 2 from the original paper was used with two exceptions: the flux through an open channel (reactions 14 and 16 in the original paper) and the diffusion of released Ca^2+^ (reaction 17) were not taken into account in order to make the model comparable with other models. This does not have an effect on the actual channel kinetics of the receptor as the removed reactions deal with Ca^2+^ flux and diffusion. Moreover, we used constant Ca^2+^ concentration and the simulated reactions happened in well-mixed system and in the present work only the kinetics of the IP_3_R, not Ca^2+^ dynamics was studied. We used the the model presented in Figure 1D and the parameter values given in Table 2.

**Table 2:** Rate constants for IP_3_R model of Dawson *et al.* [56].

Reaction	*k_f_*	*k_b_*	Reaction	*k_f_*	*k_b_*
r1	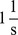	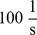	r9	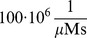	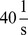
r2	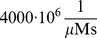	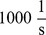	r10	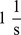	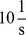
r3	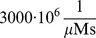	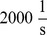	r11	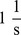	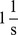
r4	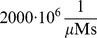	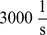	r12	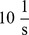	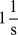
r5	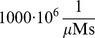	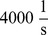	r13	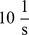	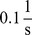
r6	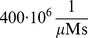	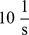	r15	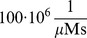	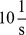
r7	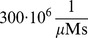	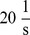	r18	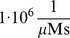	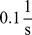
r8	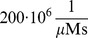	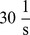	r19	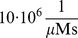	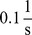

r1 to r19 refer to reactions presented in Figure 1D.

#### The model of Fraiman and Dawson

The IP_3_R model of Fraiman and Dawson [57] was originally built to study the effects of different Ca^2+^ concentrations inside the ER to the kinetics of IP_3_R. It is the only model included in the present study that has a Ca^2+^ binding site inside the ER in addition to the cytosolic binding sites. The state scheme of the model of Fraiman and Dawson [57] is presented in Figure 1C and the parameter values used in this work are in Table 3.

**Table 3:** Rate constants for IP_3_R model of Fraiman and Dawson [57], taken from [67].

Reaction	*k_f_*	*k_b_*
r1	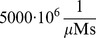	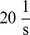
r2	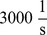	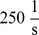
r3	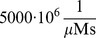	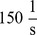
r4	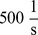	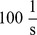
r5	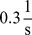	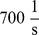
r6	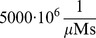	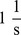
r7	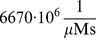	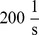
r8	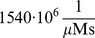	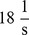
r9	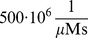	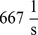
r10	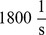	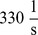
r11	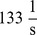	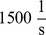
r12	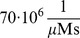	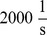
r13	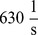	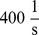
r14	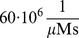	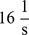

r1 to r14 refer to reactions represented in Figure 1C.

Originally, six states, O_*a*_, O_*b*_, O_*c*_, P_*a*_, P_*b*_, and P_*c*_, were considered open. However, it has been experimentally shown that IP_3_R needs IP_3_ to reach a stable open conformation [33], [62]. For this reason, we neglected three of the original open states (i.e., they were considered closed) in the present work and only states O_*a*_, O_*b*_, and O_*c*_ were considered open. In addition, in the original publication [57], the rate constant of the transition from A_10_ to A_00_ is defined as ‘detailed balance’, with no given numerical value. In our study, it was mandatory to have a numerical value for the parameter and thus we fixed the parameter by testing three values with open probability simulations (data not shown). The parameter values of 0 s^−1^ and 200 s^−1^ produced identical results which were in accordance with the results in the original publication [57], while the value of 2000 s^−1^ slightly upraised the left side of the open probability curve. Based on these simulations we chose the value of 200 s^−1^ for the transition from A_10_ to A_00_ (reaction 7, k_*b*_) and concluded that it was in the range of what was originally used.

#### The model of Doi *et al*

The IP_3_R model of Doi *et al.* [35] was originally published as part of a larger model for Ca^2+^ dynamics in the cerebellar Purkinje cell spine to investigate the role of IP_3_Rs as a coincidence detector of two input signals. Doi *et al.* [35] constructed their model based on a conceptual model of Adkins and Taylor [34]. Doi *et al.* [35] used experimental data by Khodakhah and Ogden [63], Marchant and Taylor [33], and Fujiwara *et al.* [31] to define the structure and kinetics of the model and experimental data by Bezprozvanny *et al.* [16] to test how well the model can reproduce the bell-shaped curve. A schematic representation of the model is presented in Figure 1B and the rate constants for each reaction in Table 4. In the model of Doi *et al.* [35], IP_3_R has seven states and the receptor needs to bind both IP_3_ and Ca^2+^ to open and thus provide Ca^2+^ flux from ER lumen to cytosol. In this model, IP_3_R has one open state, RIC.

**Table 4:** Rate constants for IP_3_R model of Doi *et al.* [35].

Reaction	*k_f_*	*k_b_*
r1	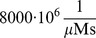	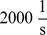
r2	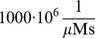	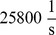
r3	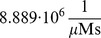	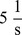
r4	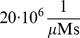	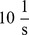
r5	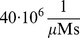	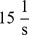
r6	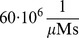	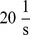

r1 to r6 refer to reactions represented in Figure 1B.

### Simulations and data analysis

In the present study, the simulations were designed to reproduce the data produced in experimental electrophysiological measurements from neuronal IP_3_Rs. We used stochastic simulation approaches since deterministic approaches were not applicable due to the stochastic nature of ion channel gating. The simulated data was compared with experimental data available in literature. The four selected models were implemented according to the information presented in the original publications with some exceptions presented in the section ‘Models’. Our work does not include parameter estimation (as, for example, [36]) since raw data on channel kinetics of IP_3_Rs in neurons is not publicly available.

In this work, STEPS (STochastic Engine for Pathway Simulation) ([58], [59]; http://steps.sourceforge.net/) version 1.1.2 was used for simulation. With STEPS, it is possible to perform full stochastic simulation of reactions and diffusion of molecules in three dimensions and also deterministic simulations. For stochastic simulations, STEPS uses the stochastic simulation algorithm (SSA) described by Gillespie [64]. The model scripts are available at ModelDB (http://senselab.med.yale.edu/ModelDB/).

In our simulations, we assumed a well-mixed system. Our models had two compartments, cytosol and ER lumen, each having volume of 0.1 fl and a surface, ER, between them. The IP_3_R was placed on the surface and the cytosolic concentrations of Ca^2+^ and IP_3_ were kept constant in the simulations to mimic the buffered conditions in patch-clamp recording.

The simulations were run on a stand-alone Linux computer. For open probability curves, simulations were repeated, depending on the model, 750–12 000 times and averaged over the repetitions for each data point. To produce one such curve, the simulations lasted from an hour to several hours. Simulations for open and closed time distributions were run once for 10–5000 s to obtain sufficient number of events to get statistically significant results. These computations took from less than a second to a couple of seconds each. Analysis of the simulated data was performed and the figures were drawn with MATLAB [65].

## Results

We compared four kinetic models previously developed for IP_3_ receptor function by simulating them with the Gillespie stochastic simulation algorithm of STEPS simulator. The comparison was done by analyzing the steady-state behavior, such as the open probability, open and closed time distributions, and the mean open and closed time. Here we show that the behavior of the models varies and some models behave somewhat unrealistically.

### Open probability

**Figure 2:**
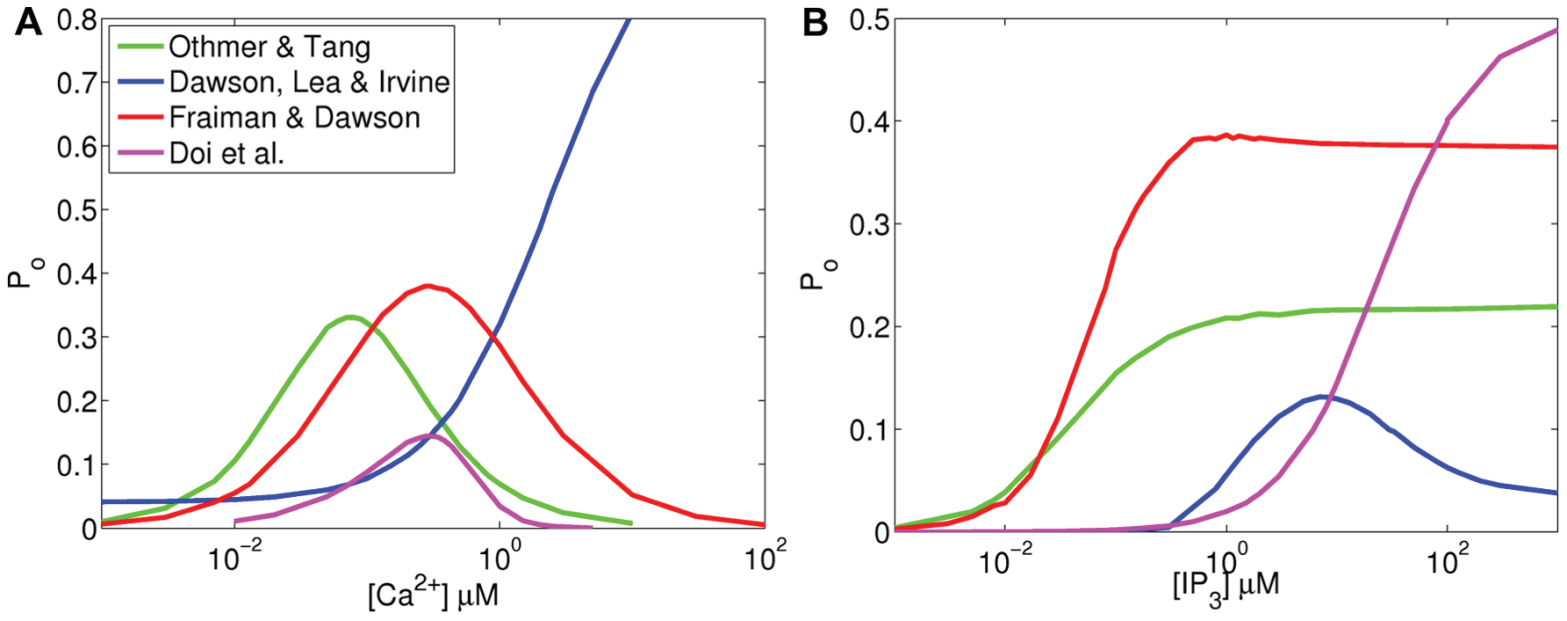
Open probability of IP_3_R as a function of (A) cytosolic Ca^2+^ concentration (IP_3_  = 10 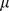
M) and (B) cytosolic IP_3_ concentration (Ca^2+^  = 0.25 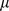
M). Green: Othmer and Tang [55], Blue: Dawson *et al.* [56], Red: Fraiman and Dawson [57], Magenta: Doi *et al.* [35].

It has been experimentally shown that the open probability (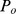) of IP_3_R is dependent on the cytosolic Ca^2+^ concentration and that the dependence is bell-shaped [16]. We repeated similar experiments by computational means and tested whether the selected four models are capable of expressing the bell-shaped curve. All the models except the model of Dawson *et al.* [56] produced the bell-shaped curve (see Figure 2A). Instead, the model of Dawson *et al.* [56] (blue in Figure 2A) produced an s-shaped curve similarly as in a previous comparison study by Sneyd *et al.* [36]. The model of Othmer and Tang [55] (green in Figure 2A) reaches the highest 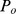 (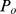 = 0.33) at cytosolic Ca^2+^ concentration around 80 nM. The model of Doi *et al.* [35] (magenta in Figure 2A) and the model of Fraiman and Dawson[57] (red in Figure 2A) reach the highest 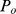 (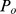  = 0.15 and 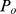  = 0.38, respectively) around [Ca^2+^]  =  300 nM, which is closest to the experimentally obtained values ([Ca^2+^]  = 250 nM by Bezprozvanny *et al.* [16] and [Ca^2+^]  =  200 nM by Kaznacheyeva *et al.* [22]). The absolute value of 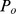 obtained in simulations cannot be directly compared to the experimental data, because Bezprozvanny *et al.* [16] and Kaznacheyeva *et al.* [22] report only normalized values, not absolute values, for 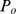.

The open probability of IP_3_R is also dependent on cytosolic IP_3_ concentration (see for example [17], [27], [29]). The open probability curves of the models obtained in simulations are shown in Figure 2B. All the models except the model of Dawson *et al.* [56] (blue in Figure 2B) follow the s-shape that is reported in experimental studies [17], [27], [29]. In their study on IP_3_Rs on Purkinje cell nuclear membrane, Marchenko *et al.* [27] have shown that the 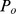 stays close to 0 until IP_3_ concentration reaches 0.3 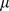
M and keeps rising until IP_3_ concentration is 3 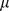
M ([Ca^2+^]  =  .25 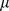
M). Watras *et al.* [17] have shown that the rise starts when IP_3_ concentration is 0.03 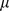
M and settles after 1 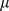
M. The 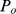 in models of Dawson *et al.* [56] (blue in Figure 2B) and Doi *et al.* [35] (magenta in Figure 2B) starts rising approximately at the same IP_3_ concentration as 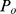 in [27], but the elevation does not stop at the right concentrations. In the models of Othmer and Tang [55] (green in Figure 2B) and Fraiman and Dawson [57] (red in Figure 2B), 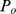 starts rising one or two orders of magnitude too low when compared to the experimental results.

**Figure 3:**
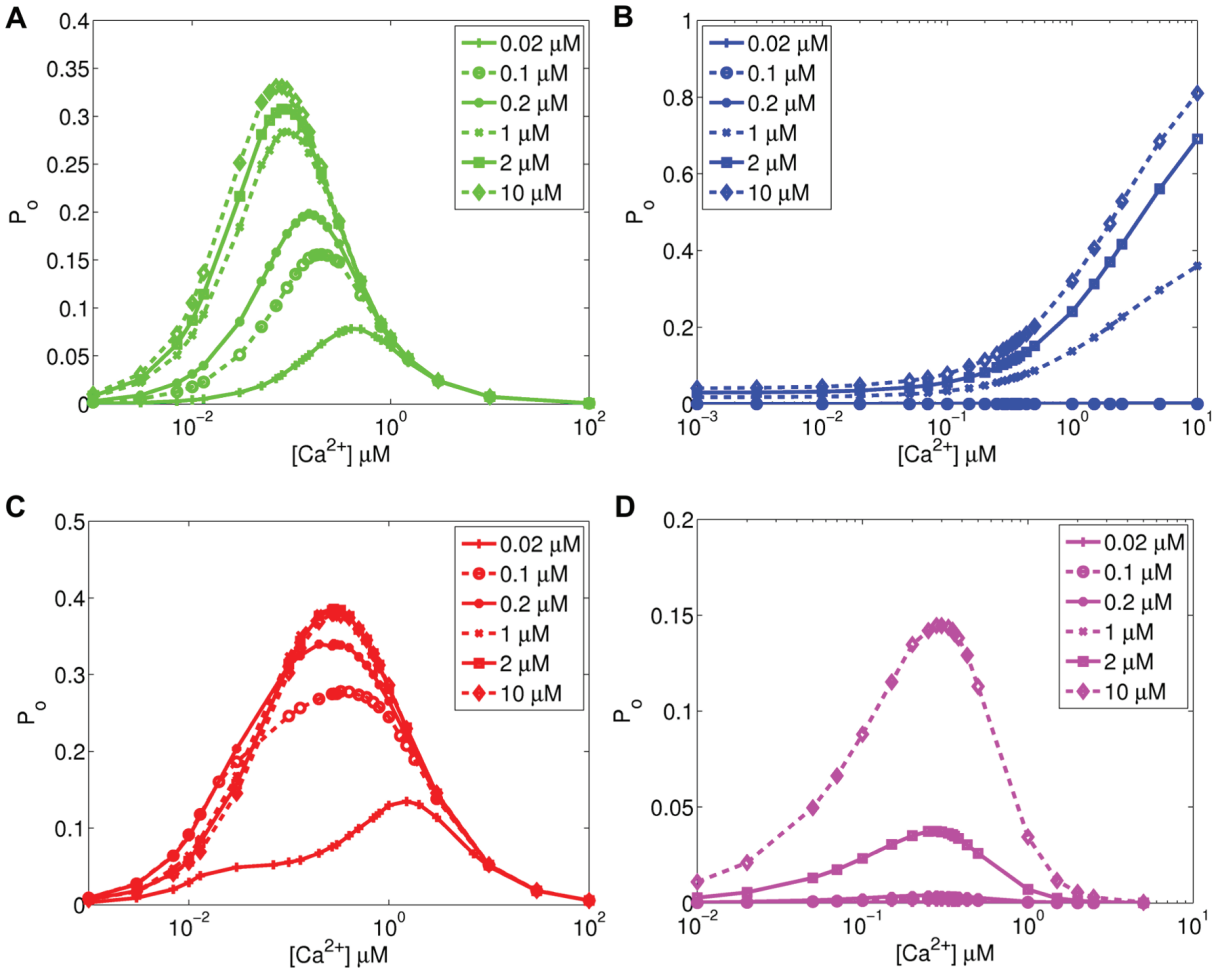
Open probability of IP_3_R as a function of cytosolic Ca^2+^ concentration in different IP_3_ concentrations. (A) Othmer and Tang [55] (B) Dawson *et al.* [56] (C) Fraiman and Dawson [57] (D) Doi *et al.* [35].

Kaftan *et al.* [19] have shown in their experiments on cerebellar IP_3_R that the bell-shaped Ca^2+^-dependence curve moves upward and to the right when IP_3_ concentration is increased. They used IP_3_ concentration values of 0.02, 0.2, 2, and 180 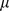
M. We used the same concentrations, in addition to their fivefold values, except 180 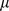
M in our simulation for all the models (results in Figure 3). The model of Othmer and Tang [55] (Figure 3A) shows a shift upward and to the left, the model of Dawson *et al.* [56] (Figure 3B) upward, and the models of Fraiman and Dawson [57] (Figure 3C) and Doi *et al.* [35] (Figure 3D) upward and slightly to the left when IP_3_ concentration increases. Similar trend has also been shown for the model of Othmer and Tang [55] by Diambra and Guisoni [66] and Tang *et al.* [43]. None of the models reproduced the results presented by Kaftan *et al.* [19].

### Mean open and closed times and distributions of open and closed times

**Figure 4:**
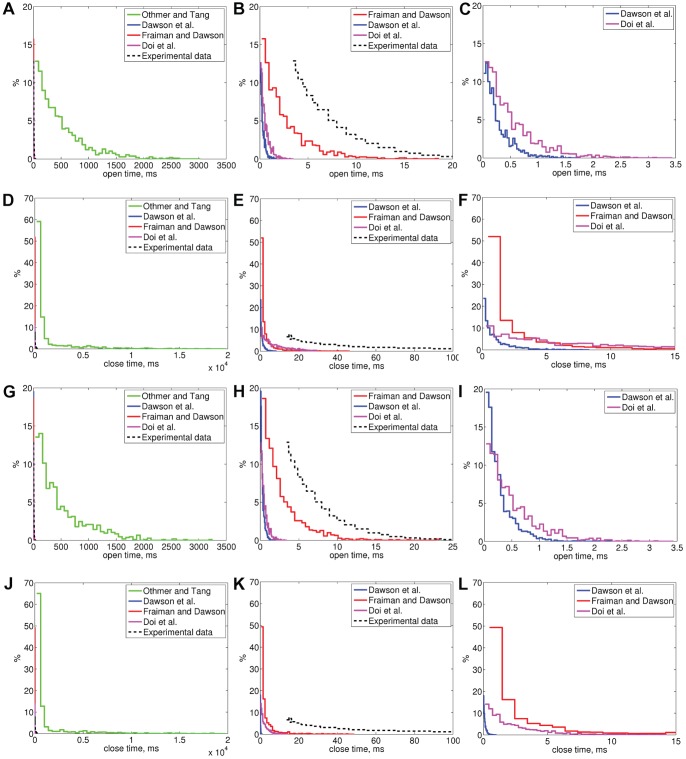
Distribution of IP_3_R open and closed times for all the selected models obtained in simulation conditions Sim 1 (A–F) and Sim 2 (G–L). (A) Open time distributions of all the models in conditions Sim 1, (B) Enlarged from A, (C) Enlarged from B, (D) Closed time distributions of all the models conditions Sim 1, (E) Enlarged from D, (F) Enlarged from E, (G) Open time distributions of all the models conditions Sim 2, (H) Enlarged from G, (I) Enlarged from H, (J) Closed time distributions of all the models conditions Sim 2, (K) Enlarged from J, (L) Enlarged from K. Experimental data is from [22]. In simulation conditions Sim 1 [Ca^2+^]  = 0.2 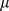
M, [IP_3_]  =  2 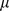
M and Sim 2 [Ca^2+^]  = 0.2 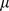
M, [IP_3_]  = 10 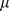
M (as shown in Table 6).

Bezprozvanny and Ehrlich [18] reported that the mean open time of canine cerebellar IP_3_R is 2.9 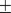 0.2 ms and Kaznacheyeva *et al.* [22] that the mean open time of wild-type rat cerebellar IP_3_R is 4.2
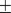
0.5 ms and that the open and closed times have exponential distributions (black dashed line in Figure 4E and 4K) in certain experimental conditions (lipid bilayer experiments, [IP_3_]  =  2 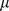
M, [Ca^2+^]  =  0.2 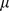
M). We simulated the selected models in these same conditions (Sim 1, results in Table 5 and Figure 4A–F) and, in order to take into account the affinity difference [31], with five times greater IP_3_ concentration (Sim 2, results in Table 5 and Figure 4G–L). The mean open times of the model of Fraiman and Dawson [57] are 2.5 ms (Sim 1) and 2.6 ms (Sim 2). These values are close to the experimentally obtained values. The mean open times obtained with the other models are an order of magnitude smaller (0.5 ms for Dawson *et al.* [56] and Doi *et al.* [35]) or significantly greater (460 ms, Othmer and Tang [55]). None of open time distributions of the selected models (Figures 4A-C and 4G-I) follow the experimental distribution by Kaznacheyeva *et al.* [22] fully, but all give, however, the exponential shape (see Figures 4B and 4K). The open time distribution of the model of Fraiman and Dawson [57] is the closest to experimentally [22] obtained distribution (see Figures 4B, 4H). The same applies also to the closed time distributions (see Figures 4E, 4K).

**Table 5:** Mean open and closed times of IP_3_R of the selected models.

	Model	mean open time (ms)	mean closed time (ms)	n	simulation time (s)
Sim 1	Othmer and Tang	451.19±423.06	12892563	1068	1 800
	Dawson *et al.*	0.59±5.46	10.33120.24	1797	20
	Fraiman and Dawson	2.45±2.52	4.0111.22	1535	10
	Doi *et al.*	0.470.46	11.2138.08	1711	20
Sim 2	Othmer and Tang	463.55463.96	12902793	1045	1 800
	Dawson *et al.*	0.524.70	9.38167.55	1897	20
	Fraiman and Dawson	2.572.76	4.9219.84	1391	10
	Doi *et al.*	0.470.46	3.7623.11	1501	6
Sim 3	Othmer and Tang	510.08526.46	1047±2074	1927	3 000
	Dawson *et al.*	0.465.90	8.83±97.56	2004	20
	Fraiman and Dawson	2.482.64	5.38±2.64	1293	10
	Doi *et al.*	0.530.53	19.00±29.60	1024	20
Sim 4	Othmer and Tang	509.68525.59	958.50±2073	2044	3 000
	Dawson *et al.*	0.6510.64	5.21±100.02	2063	10
	Fraiman and Dawson	2.512.67	5.50±15.55	1249	10
	Doi *et al.*	0.510.50	4.72±0.50	1866	10
Sim 5	Othmer and Tang	598.32598.68	3356±3384	1263	5 000
	Dawson *et al.*	0.250.25	12.60±129.80	1161	10
	Fraiman and Dawson	2.472.60	25.18±88.87	1446	40
	Doi *et al.*	0.470.47	107.37±123.06	1854	200
Sim 6	Othmer and Tang	596.98592.01	2712±2709	1509	5 000
	Dawson *et al.*	0.250.26	9.27±163.25	2098	20
	Fraiman and Dawson	2.492.61	27.54±95.62	1331	40
	Doi *et al.*	0.460.46	27.92±47.50	1407	40

The different simulation conditions (Sim 1 – Sim 6) are presented in Table 6.

**Figure 5:**
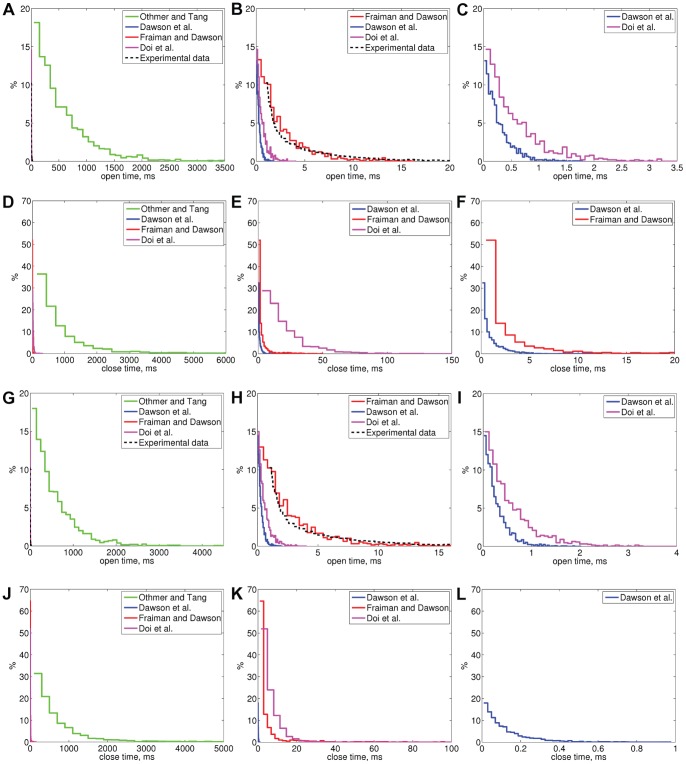
Distributions of IP_3_R open and closed times for all the selected models obtained in simulation conditions Sim 3 (A–F) and Sim 4 (G–L). (A) Open time distributions of all the models in conditions Sim 3, (B) Enlarged from A, (C) Enlarged from B, (D) Closed time distributions of all the models in conditions Sim 3, (E) Enlarged from D, (F) Enlarged from E, (G) Open time distributions of all the models conditions Sim 4, (H) Enlarged from G, (I) Enlarged from H, (J) Closed time distributions of all the models conditions Sim 4, (K) Enlarged from J, (L) Enlarged from K. Experimental data is from [20]. In simulation conditions Sim 3 [Ca^2+^] = 0.1 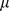
M, [IP_3_] = 2 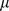
M and Sim 4 [Ca^2+^]  =  0.1 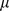
M, [IP_3_]  = 10 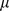
M (as shown in Table 6).

**Figure 6:**
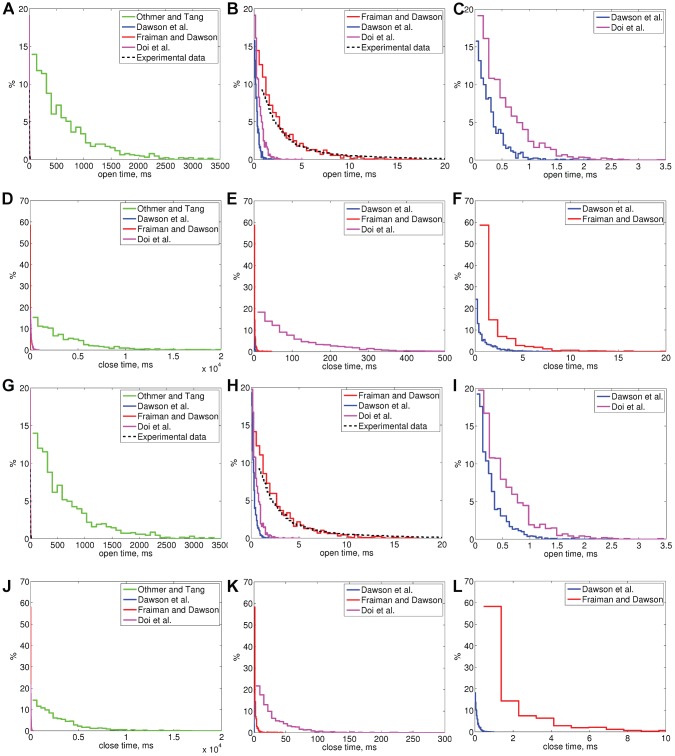
Distribution of IP_3_R open and closed times for all the selected models obtained in simulation conditions Sim 5 (A–F) and Sim 6 (G–L). (A) Open time distributions of all the models conditions Sim 5, (B) Enlarged from A, (C) Enlarged from B, (D) Closed time distributions of all the models conditions Sim 5, (E) Enlarged from D, (F) Enlarged from E, (G) Open time distributions of all the models conditions Sim 6, (H) Enlarged from G, (I) Enlarged from H, (J) Closed time distributions of all the models conditions Sim 6, (K) Enlarged from J, (L) Enlarged from K. Experimental data is from [20]. In simulation conditions Sim 5 [Ca^2+^]  = 0.01 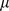
M, [IP_3_]  = 2 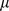
M and Sim 6 [Ca^2+^]  = 0.01 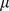
M, [IP_3_]  = 10 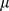
M (as shown in Table 6).

Moraru *et al.* [20] have presented open time distributions for canine cerebellar IP_3_R in two different conditions (lipid bilayer experiments, [Ca^2+^]  = 0.1 and 0.01 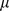
M, and [IP_3_]  = 2 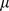
M) (black dashed line in Figures 5 and 6). We simulated the behavior of the selected models in these same experimental conditions (Sim 3 and Sim 4, results in Table 5 and Figure 5) and also with fivefold IP_3_ concentration (Sim 5 and Sim 6, results in Table 5 and Figure 6). The distributions in the wet-lab experiments are of exponential shape [18]–[20], [22] and simulation results also show exponential shape for all the models. The only distributions that are also otherwise similar to the ones obtained in wet-lab experiments by Moraru *et al.* [20] are the distributions of the model of Fraiman and Dawson [57] (Figures 5B, 5H, 6B, and 6H). All the simulation conditions used are summarized in Table 6.

**Table 6:** Ca^2+^ and IP_3_ concentration used in different simulations for open and closed time distributions.

	[Ca^2+^] (*μ*M)	[IP_3_] (*μ*M)
Sim 1	0.2	2
Sim 2	0.2	10
Sim 3	0.1	2
Sim 4	0.1	10
Sim 5	0.01	2
Sim 6	0.01	10

The simulations were done in the same conditions as wet-lab experiments [20], [22] and with five times greater IP_3_ concentration in order to take into account the affinity difference between *in vivo* and lipid bilayer experiments [31].

The Ca^2+^ concentrations used in the experiments by Moraru *et al.* [20] are unfortunately at the border or smaller than those observed in a neuron at resting level (i.e., Ca^2+^ used is 0.1 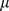
M or less). As IP_3_R is, however, known to have functional significance only above the resting level concentrations, more emphasis should be put on physiological conditions in experimental work in the future. In other words, experimental work should additionally be performed with Ca^2+^ concentrations above the known resting level.

## Discussion

In this work, four models of IP_3_R [35], [55]–[57] were selected among many to examine their steady-state and time series behavior and compare them with experimental data available in literature. We implemented and simulated the selected models using stochastic simulation software STEPS in order to obtain similar data as in single-channel patch-clamp recordings. The open probability curves and statistics, such as the mean open time and open and closed time distributions, were compared to experimental ones obtained in the same conditions. To our knowledge, this is the first detailed evaluation of IP_3_R model kinetics with stochastic methods. Our comparative study shows significant differences in the behavior and kinetics of the studied models.

Based on our results, the statistical properties of the model of Fraiman and Dawson [57] seem to be the most similar to the ones obtained in wet-lab experiments. The properties of the model of Othmer and Tang [55] are very different when compared to the experimental data. All the models except the model of Dawson *et al.* [56] produce the bell-shaped open probability curve for Ca^2+^-dependence and the s-shaped open probability curve for IP_3_-dependence as seen in the electrophysiological experiments (for example [16], [17], [27]). However, none of the models reproduce the experimental finding presented by Kaftan *et al.* [19], which shows that Ca^2+^-dependent open probability curve moves to the right and upward when IP_3_ concentration increases. This kind of behavior is shown in the original article by Fraiman and Dawson [57]. The reason why the simulation of the same model in this study did not produce similar behavior might be the slight modification that we were forced to make to the model (defining a numerical value for the one parameter that was originally defined as ‘detailed balance’ and neglecting three of the six open states). It is also notable that there is an Errata [67] published for the original article [57] and that we used the parameter set in the Errata [67].

The simulated open and closed time distributions of all the models follow the exponential distribution as does the data from experiments [18]–[20], [22]. However, the distributions are not similar apart from the distribution of Fraiman and Dawson [57]. The reason for this may be the relatively simple structure of the models, insufficiency of modeled states to reproduce the kinetics, and parameter values that do not fit the data.

According to our results, the mean open time of model of Doi *et al.* [35] is not congruent with the experimental findings. However, the shape and peak value of the open probability curve are in accordance with experimental data. As the model of Doi *et al.* [35] has originally been published as part of a larger signal transduction model for LTD induction, some inaccuracy in the behavior of the model could have been corrected by other parameters, such as the Ca^2+^ flux rate and thus the small mean open time does not invalidate the results in the original publication.

As our comparative study points out significant differences in the behavior and kinetics of the studied models, it is of interest to consider reasons for it. We identify four major reasons why the selected models behave differently to each other: 1) the structure (i.e. the equations) and parameter values differ between the models, 2) experimental data that was used in the model development vary, 3) different data handling procedures have been used when developing the models, and 4) model developers did not use automated parameter estimation methods. Next, we will discuss each issue in detail.

Firstly, the most obvious reason for differences in the behavior of models is the structure and parameter values of the models. All the models studied here have different number of states, but this does not cause the differences as such. More importantly, different parameter values and thus the affinities of IP_3_, as well as activating and inactivating Ca^2+^, vary between the models. Since the models of Othmer and Tang [55] and Doi *et al.* [35] reproduce the correct shapes for the open probability curves, re-estimation of their parameters might improve the fitting of models to experimental data. As a general conclusion, all studies neither report the values of all parameters used in simulations nor make it evident which parameter set is used to produce specific results. This makes it difficult to reproduce results (see also discussion in [68]).

Secondly, another reason for the differences in the behavior of the models could be related to the variability in the use of experimental data when constructing the original model. Although the statistical properties of channel kinetics, such as the mean open time and the distributions of open times, are known to be important in properly reconstructing receptor-ion channel kinetics, they are relatively rarely used in developing or evaluating models for IP_3_R. Furthermore, there exists a clear difference on how experimental data is used to construct (i.e., to define the structure, the number of states, and the number of parameter values in the model) and fine-tune the models (estimation of the unknown parameters). We have noticed that it is not always clear which data is used in modeling and, particularly, how it is used. In general, the models presented for IP_3_R are constructed based on only some of the data or knowledge obtained from various animal species and experiments. Furthermore, data on kinetics of IP_3_R have been obtained from various sources: native and recombinantly expressed receptors in cell lines and *Xenopus* oocytes, and from vertebrate cerebellum or hepatocytes.

Doi *et al.* [35] use the model of Adkins and Taylor [34] as their starting point and construct the model based on data by Marchant and Taylor [33] and use the open probability curve of Bezprozvanny *et al.* [16] to study the fitness of their model. The model of Othmer and Tang [55] is also shown to fit the data by Bezprozvanny *et al.* [16] in addition to data by Watras *et al.* [17] in [43], but this study does not take the difference in IP_3_ affinity [31] into account as Doi *et al.* [35] or study the open or closed time distributions of the model. Fraiman and Dawson [57] and Dawson *et al.* [56] use several experimental observations when constructing their model, but they do not report using any data for actual fitting of the model parameters. The data that Dawson *et al.* [56] compare their model to is more dealing with temporal aspect of Ca^2+^ release and accumulation of Ca^2+^ to cytosol than actual channel kinetics.

Thirdly, the differences between the simulated and experimentally observed open time distributions and mean open times might also be due to differences in data handling procedures. Experimentally observed open time distributions can be biased due to the limitations and established practices regarding the temporal resolution in the patch-clamp recordings, while in the simulations in this study all the events are recorded exactly at the time they happen. Usually the time resolution in patch-clamp recordings is around 1 ms and thus any opening shorter than that would stay unnoticed or be merged with other channel openings.

Fourthly, to our knowledge, automated parameter estimation methods have not been used in the development of the four models here compared. Studies on IP_3_R models consider, to some extent, the kinetic ion channel data to define the mathematical structure of the models. However, only a few previous studies use automated parameter estimation techniques and statistical data on ion channel kinetics to fine-tune the IP_3_R models [36], [69]–[72].

One of the major challenges in modeling the IP_3_Rs is the lack of access to original raw data, for example from electrophysiological measurements, that could be used in quantitative modeling. This data is not currently available in any public database and as the years pass by it becomes extremely hard to acquire the data from its original sources. This problem is not new or limited just to measurements of ion channels but to all neuroscience data [73], [74]. Some suggestions to improve the situation have been made. For instance, De Schutter [75] suggests that data publishing should be distinguished from paper publishing. Furthermore, Ranjan *et al.* [76] have established an information management framework for ion channel information, which hopefully will make IP_3_R experimental data more accessible in the future.

Despite several shortcomings in the development and presentation of models, previous models on IP_3_R, including the present comparative study on four stochastic IP_3_R models, will give a good setting for constructing a new, realistic model of IP_3_Rs for compartmental modeling of neuronal functions. It will be a challenge to develop computationally inexpensive models that can produce realistic stochastic behavior of an individual ion channel. A wealth of evidence indicates, however, an important role of randomly opening ion channels on the global behavior of cells. For example, in neurons the stochastic openings of single ion channels shape the integration of local signals in dendrites or spines [77], stochastic openings of voltage-gated ion channels have an important role in adjusting the transmembrane voltage dynamics [78]–[80], and the reliability of action potential propagation along thin axons is affected by the stochastic opening of voltage-gated ion channels [81]. Furthermore, molecular noise of single ion channel is shown to be translated into global cellular processes in astrocytes [82].

In summary, the development of new IP_3_R models clearly calls for both steady-state and kinetic data. Fitting of the new computational models should be done using automated estimation techniques, possibly using Bayesian approaches [72], [83]–[85]. Data for model construction and fine-tuning would ideally be acquired from the same neuronal type as the model is built for.

## References

[1] Libersat F, Duch C (2004) Mechanisms of dendritic maturation. Mol Neurobiol 29: 303–320. 15181241 10.1385/MN:29:3:303

[2] Michaelsen K, Lohmann C (2010) Calcium dynamics at developing synapses: mechanisms and functions. Eur J Neurosci 32: 218–223. 20646046 10.1111/j.1460-9568.2010.07341.x

[3] Banerjee S, Hasan G (2005) The InsP_3_ receptor: its role in neuronal physiology and neurodegeneration. Bioessays 27: 1035–1047. 16163728 10.1002/bies.20298

[4] Foskett J (2010) Inositol trisphosphate receptor Ca^2+^ release channels in neurological diseases. Pflugers Arch Eur J Physiol 460: 481–494. 20383523 10.1007/s00424-010-0826-0 PMC2893360

[5] Bliss T, Collingridge G (1993) A synaptic model of memory: long-term potentiation in the hippocampus. Nature 361: 31–39. 8421494 10.1038/361031a0

[6] Franks KM, Sejnowski TJ (2002) Complexity of calcium signaling in synaptic spines. BioEssays 24: 1130–1144. 12447978 10.1002/bies.10193 PMC2944017

[7] Ogasawara H, Doi T, Kawato M (2008) Systems biology perspectives on cerebellar long-term depression. Neurosignals 16: 300–317. 18635946 10.1159/000123040

[8] Collingridge G, Peineau S, Howland J, Wang Y (2010) Long-term depression in the CNS. Nature Rev Neurosci 11: 459–473. 20559335 10.1038/nrn2867

[9] Citri A, Malenka R (2008) Synaptic plasticity: multiple forms, functions, and mechanisms. Neuropsychopharmacology 33: 18–41. 17728696 10.1038/sj.npp.1301559

[10] Sharp A, Nucifora Jr F, Blondel O, Sheppard C, Zhang C, et al (1999) Differential cellular expression of isoforms of inositol 1,4,5-triphosphate receptors in neurons and glia in brain. J Comp Neurol 406: 207–220. 10096607

[11] Ito M (2002) The molecular organization of cerebellar long-term depression. Nature Rev Neurosci 3: 896–902. 12415297 10.1038/nrn962

[12] Foskett JK, White C, Cheung KH, Mak DOD (2007) Inositol trisphosphate receptor Ca^2+^ release channels. Physiol Rev 87: 593–658. 17429043 10.1152/physrev.00035.2006 PMC2901638

[13] Llano I, DiPolo R, Marty A (1994) Calcium-induced calcium release in cerebellar Purkinje cells. Neuron 12: 663–673. 7512352 10.1016/0896-6273(94)90221-6

[14] Barbara J (2002) IP_3_-dependent calcium-induced calcium release mediates bidirectional calcium waves in neurones: functional implications for synaptic plasticity. Biochim Biophys Acta – Proteins & Proteomics 1600: 12–18. 10.1016/s1570-9639(02)00439-9 12445454

[15] Ehrlich B, Watras J (1988) Inositol 1,4,5-trisphosphate activates a channel from smooth muscle sarcoplasmic reticulum. Nature 336: 583–586. 2849060 10.1038/336583a0

[16] Bezprozvanny I, Watras J, Ehrlich B (1991) Bell-shaped calcium-response curves of Ins(1,4,5)P_3_- and calcium-gated channels from endoplasmic reticulum of cerebellum. Nature 351: 751–754. 1648178 10.1038/351751a0

[17] Watras J, Bezprozvanny I, Ehrlich B (1991) Inositol 1,4,5-trisphosphate-gated channels in cerebellum: presence of multiple conductance states. J Neurosci 11: 3239. 1719158 10.1523/JNEUROSCI.11-10-03239.1991 PMC6575433

[18] Bezprozvanny I, Ehrlich B (1994) Inositol (1,4,5)-trisphosphate (InsP_3_)-gated Ca channels from cerebellum: conduction properties for divalent cations and regulation by intraluminal calcium. J Gen Phys 104: 821–856. 10.1085/jgp.104.5.821 PMC2229238 7876825

[19] Kaftan E, Ehrlich B, Watras J (1997) Inositol 1,4,5-trisphosphate (InsP_3_) and calcium interact to increase the dynamic range of InsP3 receptor-dependent calcium signaling. J Gen Physiol 110: 529–538. 9348325 10.1085/jgp.110.5.529 PMC2229389

[20] Moraru I, Kaftan E, Ehrlich B, Watras J (1999) Regulation of type 1inositol 1,4,5-trisphosphategated calcium channels by InsP_3_ and calcium. Simulation of single shannel kinetics based on ligand binding and electrophysiological analysis. J Gen Physiol 113: 837–849. 10352034 10.1085/jgp.113.6.837 PMC2225610

[21] Maeda N, Kawasaki T, Nakade S, Yokota N, Taguchi T, et al (1991) Structural and functional characterization of inositol 1,4,5-trisphosphate receptor channel from mouse cerebellum. J Biol Chem 266: 1109–1116. 1845986

[22] Kaznacheyeva E, Lupu VD, Bezprozvanny I (1998) Single-channel properties of inositol (1,4,5)- trisphosphate receptor heterologously expressed in HEK-293 cells. J Gen Physiol 111: 847–856. 9607940 10.1085/jgp.111.6.847 PMC2217157

[23] Dellis O, Dedos S, Tovey S, Taufiq-Ur-Rahman, Dubel S, et al (2006) Ca^2+^ entry through plasma membrane IP_3_ receptors. Science 313: 229. 16840702 10.1126/science.1125203

[24] Wagner I, Larry E, Joseph S, Yule D (2008) Regulation of single inositol 1,4,5-trisphosphate receptor channel activity by protein kinase a phosphorylation. J Physiol 586: 3577–3596. 18535093 10.1113/jphysiol.2008.152314 PMC2538833

[25] Wagner I, Larry E, Yule D (2012) Differential regulation of the InsP_3_ receptor type-1 and -2 single channel properties by InsP_3_, Ca^2+^ and ATP. J Physiol 590: 3245–3259. 22547632 10.1113/jphysiol.2012.228320 PMC3459040

[26] Mak D, Foskett J (1994) Single-channel inositol 1,4,5-trisphosphate receptor currents revealed by patch clamp of isolated Xenopus oocyte nuclei. J Biol Chem 269: 29375–29378. 7961913

[27] Marchenko S, Yarotskyy V, Kovalenko T, Kostyuk P, Thomas R (2005) Spontaneously active and InsP_3_-activated ion channels in cell nuclei from rat cerebellar Purkinje and granule neurones. J Physiol 565: 897–910. 15774532 10.1113/jphysiol.2004.081299 PMC1464565

[28] Marchenko S, Thomas R (2006) Nuclear Ca^2+^ signalling in cerebellar Purkinje neurons. The Cerebellum 5: 36–42. 16527762 10.1080/14734220600554438

[29] Taufiq-Ur-Rahman Skupin A, Falcke M, Taylor C (2009) Clustering of InsP_3_ receptors by InsP_3_ retunes their regulation by InsP_3_ and Ca^2+^ . Nature 458: 655–659. 19348050 10.1038/nature07763 PMC2702691

[30] Mak D, McBride S, Foskett J (2001) ATP regulation of recombinant type 3 inositol 1, 4, 5- trisphosphate receptor gating. J Gen Physiol 117: 447–456. 11331355 10.1085/jgp.117.5.447 PMC2233659

[31] Fujiwara A, Hirose K, Yamazawa T, Iino M (2001) Reduced IP_3_ sensitivity of IP_3_ receptor in Purkinje neurons. Neuroreport 12: 2647–2651. 11522941 10.1097/00001756-200108280-00012

[32] Dufour J, Arias I, Turner T (1997) Inositol 1,4,5-trisphosphate and calcium regulate the calcium channel function of the hepatic inositol 1,4,5-trisphosphate receptor. J Biol Chem 272: 2675–2681. 9006903 10.1074/jbc.272.5.2675

[33] Marchant JS, Taylor CW (1997) Cooperative activation of IP_3_ receptors by sequential binding of IP_3_ and Ca^2+^ safeguards against spontaneous activity. Curr Biol 7: 510–518. 9210378 10.1016/s0960-9822(06)00222-3

[34] Adkins C, Taylor C (1999) Lateral inhibition of inositol 1,4,5-trisphosphate receptors by cytosolic Ca^2+^ . Curr Biol 9: 1115–1118. 10531009 10.1016/s0960-9822(99)80481-3

[35] Doi T, Kuroda S, Michikawa T, Kawato M (2005) Inositol 1,4,5-trisphosphate-dependent Ca^2+^ threshold dynamics detect spike timing in cerebellar Purkinje cells. J Neurosci 25: 950–961. 15673676 10.1523/JNEUROSCI.2727-04.2005 PMC6725626

[36] Sneyd J, Falcke M, Dufour J, Fox C (2004) A comparison of three models of the inositol trisphosphate receptor. Prog Biophys Mol Biol 85: 121–140. 15142740 10.1016/j.pbiomolbio.2004.01.013

[37] Eungdamrong N, Iyengar R (2004) Modeling cell signaling networks. Biol Cell 96: 355–362. 15207904 10.1016/j.biolcel.2004.03.004 PMC3620715

[38] Hellgren Kotaleski J, Blackwell K (2010) Modelling the molecular mechanisms of synaptic plasticity using systems biology approaches. Nat Rev Neurosci 11: 239–251. 20300102 10.1038/nrn2807 PMC4831053

[39] Sneyd J, Falcke M (2005) Models of the inositol trisphosphate receptor. Prog Biophys Mol Biol 89: 207–245. 15950055 10.1016/j.pbiomolbio.2004.11.001

[40] Falcke M (2003) On the role of stochastic channel behavior in intracellular Ca^2+^ dynamics. Biophys J 84: 42–56. 12524264 10.1016/S0006-3495(03)74831-0 PMC1302592

[41] De Young G, Keizer J (1992) A single-pool inositol 1,4,5-trisphosphate-receptor-based model for agonist-stimulated oscillations in Ca^2+^ concentration. Proc Natl Acad Sci USA 89: 9895–9899. 1329108 10.1073/pnas.89.20.9895 PMC50240

[42] LeBeau A, Yule D, Groblewski G, Sneyd J (1999) Agonist-dependent phosphorylation of the inositol 1, 4, 5-trisphosphate receptor. J Gen Physiol 113: 851. 10352035 10.1085/jgp.113.6.851 PMC2225599

[43] Tang Y, Stephenson J, Othmer H (1996) Simplification and analysis of models of calcium dynamics based on IP_3_-sensitive calcium channel kinetics. Biophys J 70: 246–263. 8770202 10.1016/S0006-3495(96)79567-X PMC1224924

[44] Mak D, McBride S, Foskett J (2003) Spontaneous channel activity of the inositol 1,4,5-trisphosphate (InsP_3_) receptor (InsP_3_R). Application of allosteric modeling to calcium and InsP_3_ regulation of InsP_3_R single-channel gating. J Gen Physiol 122: 583. 14581584 10.1085/jgp.200308809 PMC2229577

[45] Shuai JW, Yang DP, Pearson JE, Rüdiger S (2009) An investigation of models of the IP_3_R channel in Xenopus oocyte. Chaos 19: 037105. 19792030 10.1063/1.3156402 PMC2771705

[46] Schuster S, Marhl M, Hofer T (2002) Modelling of simple and complex calcium oscillations. Eur J Biochem 269: 1333–1355. 11874447 10.1046/j.0014-2956.2001.02720.x

[47] Turner TE, Schnell S, Burrage K (2004) Stochastic approaches for modelling in vivo reactions. Comp Biol Chem 28: 165–178. 10.1016/j.compbiolchem.2004.05.001 15261147

[48] Barrio M, Burrage K, Leier A, Tian T (2006) Oscillatory regulation of Hes1: discrete stochastic delay modelling and simulation. PLOS Comp Biol 2: e117. 10.1371/journal.pcbi.0020117 PMC1560403 16965175

[49] Hituri K, Achard P, Wils S, Linne ML, De Schutter E (2008) Stochastic modeling of inositol-1,4,5- trisphophate receptors in Purkinje cell spine. In: Proceedings of the 5th TICSP Workshop on Computation Systems Biology (WCSB 2008). Leipzig, Germany, pp. 57–60.

[50] Choi T, Maurya M, Tartakovsky D, Subramaniam S (2010) Stochastic hybrid modeling of intracellular calcium dynamics. J Chem Phys 133: 165101. 21033822 10.1063/1.3496996 PMC2998048

[51] Gillespie DT (1976) A general method for numerical simulating the stochastic time evolution of coupled chemical reactions. J Comp Phys 22: 403–434.

[52] Sneyd J, Dufour J (2002) A dynamic model of the type-2 inositol trisphosphate receptor. Proc Natl Acad Sci USA 99: 2398–2403. 11842185 10.1073/pnas.032281999 PMC122376

[53] Swillens S, Champeil P, Combettes L, Dupont G (1998) Stochastic simulation of a single inositol 1,4,5-trisphosphate-sensitive Ca^2+^ channel reveals repetitive openings during 'blip-like' Ca^2+^ transients. Cell calcium 23: 291–302. 9681192 10.1016/s0143-4160(98)90025-2

[54] Haeri H, Hashemianzadeh S, Monajjemi M (2007) A kinetic Monte Carlo simulation study of inositol 1,4,5-trisphosphate receptor (IP_3_R) calcium release channel. Comp Biol Chem 31: 99–109. 10.1016/j.compbiolchem.2007.02.009 17392027

[55] Othmer HG, Tang Y (1993) Oscillations and waves in a model of InsP3-controlled calcium dynamics, London: Plenum Press, volume 259 of Experimental and Theoretical Advances in Biological Pattern Formation. pp. 277–300.

[56] Dawson A, Lea E, Irvine R (2003) Kinetic model of the inositol trisphosphate receptor that shows both steady-state and quantal patterns of Ca^2+^ release from intracellular stores. Biochem J 370: 621. 12479792 10.1042/BJ20021289 PMC1223205

[57] Fraiman D, Dawson SP (2004) A model of IP_3_ receptor with a luminal calcium binding site: stochastic simulations and analysis. Cell Calcium 35: 403–413. 15003850 10.1016/j.ceca.2003.10.004

[58] Wils S, De Schutter E (2009) STEPS: Modeling and simulating complex reaction-diffusion systems with Python. Front Neuroinform 3: 165–178. 10.3389/neuro.11.015.2009 PMC2706651 19623245

[59] Hepburn I, Chen W, Wils S, De Schutter E (2012) STEPS: efficient simulation of stochastic reaction-diffusion models in realistic morphologies. BMC Syst Biol 6: 1752–0509. 10.1186/1752-0509-6-36 PMC3472240 22574658

[60] Mishra J, Bhalla U (2002) Simulations of inositol phosphate metabolism and its interaction with InsP3-mediated calcium release. Biophys J 83: 1298–1316. 12202356 10.1016/S0006-3495(02)73901-5 PMC1302229

[61] Sachs F, Qin F, Palade P (1995) Models of Ca^2+^ release channel adaptation. Science 267: 2010–2011. 7701327 10.1126/science.7701327

[62] Taylor CW, da Fonseca PC, Morris EP (2004) IP_3_ receptors: the search for structure. Trends Biochem Sci 29: 210–219. 15082315 10.1016/j.tibs.2004.02.010

[63] Khodakhah K, Ogden D (1995) Fast activation and inactivation of inositol trisphosphate-evoked Ca^2+^ release in rat cerebellar Purkinje neurones. J Physiol 487: 343. 8558468 10.1113/jphysiol.1995.sp020884 PMC1156577

[64] Gillespie DT (1977) Exact stochastic simulation of coupled chemical reactions. J Phys Chem 81: 2340–2361.

[65] MATLAB (2011) version 7.13.0.564 (R2011b). Natick, Massachusetts: The MathWorks Inc.

[66] Diambra L, Guisoni N (2005) Modeling stochastic Ca^2+^ release from a cluster of IP_3_-sensitive receptors. Cell Calcium 37: 321–332. 15755493 10.1016/j.ceca.2004.12.001

[67] Fraiman D, Dawson SP (2004) Erratum to ”a model of IP_3_ receptor with a luminal calcium binding site: stochastic simulations and analysis”. Cell Calcium 36: 445. 10.1016/j.ceca.2003.10.004 15003850

[68] De Schutter E (2008) Why are computational neuroscience and systems biology so separate? PLOS Comp Biol 4: e1000078. 10.1371/journal.pcbi.1000078 PMC2367448 18516226

[69] Gin E, Falcke M, Wagner L, Yule D, Sneyd J (2009) Markov chain Monte Carlo fitting of singlechannel data from inositol trisphosphate receptors. J Theor Biol 257: 460–474. 19168073 10.1016/j.jtbi.2008.12.020

[70] Gin E, Falcke M, Wagner L, et al (2009) A kinetic model of the inositol trisphosphate receptor based on single-channel data. Biophysical journal 96: 4053. 19450477 10.1016/j.bpj.2008.12.3964 PMC2712151

[71] Gin E, Wagner L, Yule D, Sneyd J (2009) Inositol trisphosphate receptor and ion channel models based on single-channel data. Chaos 19: 037104. 19792029 10.1063/1.3184540 PMC5848693

[72] Siekmann I, Wagner L, Yule D, Fox C, Bryant D, et al (2011) MCMC estimation of Markov models for ion channels. Biophys J 100: 1919–1929. 21504728 10.1016/j.bpj.2011.02.059 PMC3077709

[73] Amari S, Beltrame F, Bjaalie J, Dalkara T, De Schutter E, et al (2002) Neuroinformatics: the integration of shared databases and tools towards integrative neuroscience. J Integr Neurosci 1: 117–128. 15011281 10.1142/s0219635202000128

[74] Cannon R, Howell F, Goddard N, De Schutter E (2002) Non-curated distributed databases for experimental data and models in neuroscience. Network: Computation in Neural Systems 13: 415–428. 12222822

[75] De Schutter E (2010) Data publishing and scientific journals: The future of the scientific paper in a world of shared data. Neuroinformatics : 1–3. 10.1007/s12021-010-9084-8 20835853

[76] Ranjan R, Khazen G, Gambazzi L, Ramaswamy S, Hill S, et al. (2011) Channelpedia: an integrative and interactive database for ion channels. Front Neuroinform 5. 10.3389/fninf.2011.00036 PMC3248699 22232598

[77] Cannon R, O'Donnell C, Nolan M (2010) Stochastic ion channel gating in dendritic neurons: Morphology dependence and probabilistic synaptic activation of dendritic spikes. PLOS Comp Biol 6: e1000886. 10.1371/journal.pcbi.1000886 PMC2920836 20711353

[78] White J, Klink R, Alonso A, Kay A (1998) Noise from voltage-gated ion channels may influence neuronal dynamics in the entorhinal cortex. J Neurophysiol 80: 262. 9658048 10.1152/jn.1998.80.1.262

[79] Steinmetz P, Manwani A, Koch C, London M, Segev I (2000) Subthreshold voltage noise due to channel fluctuations in active neuronal membranes. J Comput Neurosci 9: 133–148. 11030518 10.1023/a:1008967807741

[80] Saarinen A, Linne ML, Yli-Harja O (2008) Stochastic differential equation model for cerebellar granule cell excitability. PLOS Comp Biol 4(2): e1000004. 10.1371/journal.pcbi.1000004 PMC2265481 18463700

[81] Faisal A, Laughlin S (2007) Stochastic simulations on the reliability of action potential propagation in thin axons. PLOS Comp Biol 3: e79. 10.1371/journal.pcbi.0030079 PMC1864994 17480115

[82] Skupin A, Kettenmann H, Falcke M (2010) Calcium signals driven by single channel noise. PLOS Comp Biol 6: e1000870. 10.1371/journal.pcbi.1000870 PMC2917103 20700497

[83] Wilkinson D (2007) Bayesian methods in bioinformatics and computational systems biology. Brief Bioinform 8: 109. 17430978 10.1093/bib/bbm007

[84] Girolami M (2008) Bayesian inference for differential equations. Theor Comput Sci 408: 4–16.

[85] Penny W Stephan K, Daunizeau J, Rosa M, Friston K, et al (2010) Comparing families of dynamic causal models. PLOS Comp Biol 6: e1000709. 10.1371/journal.pcbi.1000709 PMC2837394 20300649

