# Correction: Comparison of Models for IP_3_ Receptor Kinetics Using Stochastic Simulations

**DOI:** 10.1371/annotation/b345b891-8a99-4a0f-826e-9cd17669817f

**Published:** 2013-12-13

**Authors:** Katri Hituri, Marja-Leena Linne

In Tables 1, 2, 3, and 4 the unit should be (Ms)^-1^ instead of (µMs)^-1^.

In Table 5, an error occurred during the production process involving columns 3 and 4. Please see the corrected table 5 here: http://plosone.org/corrections/pone.0059618.t005.cn.tif

